# Combined Impact of No-Till and Cover Crops with or without Short-Term Water Stress as Revealed by Physicochemical and Microbiological Indicators

**DOI:** 10.3390/biology10010023

**Published:** 2021-01-01

**Authors:** Eren Taskin, Roberta Boselli, Andrea Fiorini, Chiara Misci, Federico Ardenti, Francesca Bandini, Lorenzo Guzzetti, Davide Panzeri, Nicola Tommasi, Andrea Galimberti, Massimo Labra, Vincenzo Tabaglio, Edoardo Puglisi

**Affiliations:** 1 Dipartimento di Scienze e Tecnologie Alimentari per la sostenibilità della filiera agro-alimentare (DISTAS), Facoltà di Scienze Agrarie Alimentari ed Ambientali, Università Cattolica del Sacro Cuore, 29122 Piacenza, Italy; eren.taskin@unicatt.it (E.T.); chiara.misci1@unicatt.it (C.M.); francesca.bandini@unicatt.it (F.B.); edoardo.puglisi@unicatt.it (E.P.); 2 Dipartimento di Scienze delle Produzioni Vegetali Sostenibili (DI.PRO.VE.S.), Facoltà di Scienze Agrarie Alimentari ed Ambientali, Università Cattolica del Sacro Cuore, 29122 Piacenza, Italy; roberta.boselli@unicatt.it (R.B.); andrea.fiorini@unicatt.it (A.F.); federico.ardenti@unicatt.it (F.A.); 3 Dipartimento di Biotecnologie e Bioscienze (BtBs), Università degli Studi di Milano-Bicocca, 20126 Milano, Italy; lorenzo.guzzetti@unimib.it (L.G.); davide.panzeri@unimib.it (D.P.); nicola.tommasi@unimib.it (N.T.); andrea.galimberti@unimib.it (A.G.); massimo.labra@unimib.it (M.L.)

**Keywords:** soil bacterial community, soil fungal community, soil C and N pools, no-till, water stress

## Abstract

**Simple Summary:**

Farming systems in which no-till (NT) and cover crops (CC) are preferred as alternatives to conventional practices have the promise of being more resilient and climate smart. Our field study aimed to assess the long-term impact of NT plus CC, with vs. without short-term water stress, on soil microbial biodiversity, enzymatic activities, and the distribution of C and N pools within soil aggregates. We found that the diversity of bacteria and fungi in the soil was positively affected by NT + CC, especially under water stress conditions. Under NT + CC, the presence of important plant growth-promoting rhizobacteria was revealed. Soil enzymatic activity confirmed the depleting impact of conventional tillage. Soil C and N were increased under NT + CC due to their inclusion into large soil aggregates that are beneficial for long-term C and N stabilization in soils. Water stress was found to have detrimental effects on aggregates formation and limited C and N inclusion within aggregates. The microbiological and physicochemical parameters correlation supported the hypothesis that long-term NT + CC is a valuable strategy for sustainable agroecosystems, due to its contribution to soil C and N stabilization while enhancing the biodiversity and enzymes.

**Abstract:**

Combining no-till and cover crops (NT + CC) as an alternative to conventional tillage (CT) is generating interest to build-up farming systems’ resilience while promoting climate change adaptation in agriculture. Our field study aimed to assess the impact of long-term NT + CC management and short-term water stress on soil microbial communities, enzymatic activities, and the distribution of C and N within soil aggregates. High-throughput sequencing (HTS) revealed the positive impact of NT + CC on microbial biodiversity, especially under water stress conditions, with the presence of important rhizobacteria (e.g., *Bradyrhizobium* spp.). An alteration index based on soil enzymes confirmed soil depletion under CT. C and N pools within aggregates showed an enrichment under NT + CC mostly due to C and N-rich large macroaggregates (LM), accounting for 44% and 33% of the total soil C and N. Within LM, C and N pools were associated to microaggregates within macroaggregates (mM), which are beneficial for long-term C and N stabilization in soils. Water stress had detrimental effects on aggregate formation and limited C and N inclusion within aggregates. The microbiological and physicochemical parameters correlation supported the hypothesis that long-term NT + CC is a promising alternative to CT, due to the contribution to soil C and N stabilization while enhancing the biodiversity and enzymes.

## 1. Introduction

Conventional managements of agroecosystems based on intensive agricultural practices have often depleted soil structure and altered processes involved in the maintenance of soil fertility and provision of ecosystem services [1]. The combination of no-till and cover crops (NT + CC) as an alternative management to the conventional tillage (CT) approach is generating considerable interest to build-up farming systems resilience and to promote the climate change adaptation of agriculture [2,3,4]. Combining NT + CC with the cultivation of crops of low-water and -nutrient needs was suggested among the sustainable solutions for food security as an alternative to systems based on the monoculture of maize or other staples [5]. Microbial diversity is pivotal for the nutrient cycles and for the provision of numerous ecosystem services in soil [6,7,8,9]. Especially under challenging conditions, such as water stress, the biodiversity of soil microbial communities can act as an insurance of the functionality of terrestrial ecosystems [10,11]. Tillage could cause changes in the soil microbial communities via its impact on the physical relocation of nutrients available at different soil depths [12], soil properties [13], and residue management [14]. Furthermore, the activities of soil β-glucosidase, phosphatase, and urease were previously found to be suitable for monitoring the alteration status of soils subjected to different management [15,16]. Particularly, Alteration Index 3 (AI3), proposed by Puglisi et al. [16], integrates the activities of these three enzymes to assess the impact of agricultural practices on soil, revealing the alteration status of soil as an early indicator of its depletion.

The impact of tillage on soil structure and stability is known, but previous studies show conflicting results regarding the role of water supply on governing combined dynamics of aggregates, carbon, and nitrogen in soil. According to Trost et al. [17], sprinkler irrigation, as well as heavy rains, may fracture soil aggregates in the shallow soil layer due to water droplet impacts, and macroaggregates are more affected by breakdown due to irrigation than microaggregates [18]. On the other hand, the damaging effect of irrigation on aggregate stability may be counteracted by the increase of root and microbial biomass promoted by the optimum water availability during the growing season [19,20,21,22]. Six et al. [18] suggested also that intense tillage operations compromise the physical stabilization of most soil organic carbon (C) and nitrogen (N) within soil macroaggregates (diameters > 250 µm) by showing that the rate of macroaggregate turnover is doubled under CT compared with no-till (NT) soils. This, in turn, decreases the mean residence time of soil C by almost 50%, with detrimental effects on long-term C sequestration in agricultural soils. In contrast, NT + CC was shown to enhance soil fertility, stabilize soil structure, and increase soil C and N storage [23,24,25,26,27]. However, NT sometimes lead only to a soil C redistribution rather than a net C storage increase [28]. In addition, these effects should be evaluated in the long term, and the potential of NT for soil C and N accumulation in stable pools has already been argued [29]. The assessment of soil microbial diversity and soil enzymes, together with soil C and N dynamics and agronomic performances of studied crops, can reveal their true potential under challenging conditions for NT + CC agriculture [30].

The present field study investigated the impact of long-term CT vs. NT + CC management and short-term water stress on (i) soil bacterial and fungal communities, (ii) soil alteration through enzymatic activities, and (iii) soil C and N distribution within soil aggregates. We hypothesized that the long-term NT + CC management (i) could positively contribute to the resilience of soil bacterial and fungal communities and to soil enzymes, especially when subjected to water stress conditions, and (ii) it would enhance C and N stabilization within aggregate-sized soil fractions.

## 2. Materials and Methods

### 2.1. Field and Experimental Conditions

The study was conducted on an eight-year field experiment at the Centro Ricerche Zootecniche—CERZOO experimental farm in Piacenza (45°00′18.0′′ N, 9°42′12.7′′ E; 68 m a.s.l.), Po Valley, Northern Italy. The soil at the field site is a fine, mixed, mesic, Udertic Haplustalf (Soil Survey Staff, 2014), with a silty-clay texture (sand 122, silt 462, and clay 416 g kg^−1^) in the 0–30-cm soil layer. Main physicochemical properties of soil are reported in Fiorini et al. [20]. The climate is temperate; mean annual temperature and rainfall are 12.2 °C and 890 mm, respectively. The field study was established as a Randomized Complete Block (RCB) with four replicates and two treatments: CT and NT + CC. Cover cropping was made by sowing a mixture of rye (*Secale cereale* L.) (55%), hairy vetch (*Vicia villosa* Roth.) (25%), crimson clover (*Trifolium incarnatum* L.) (8%), Italian ryegrass (*Lolium multiflorum* Lam.) (8%), and tillage radish (*Raphanus sativus* var. *longipinnatus* L.H. Bailey) (4%) right after harvesting the previous main crop, as detailed in Boselli et al. [31]. The seedbed preparation under CT treatment consisted of a conventional ploughing at 35-cm depth during fall season, and two rotating harrowing at 15–20-cm depths before seeding. Under NT + CC treatment, the cover crop cycle was terminated in the spring by spraying Glyphosate (2.4 L ha^−1^), and two weeks after, the main crop was directly sown on no-tilled soil. Each plot was 22-m wide and 65-m long (1430 m^2^). All plots were tilled conventionally before starting NT management (2011). Between 2011 and 2018, the 7-year crop sequence was the same under both tillage treatments (CT and NT) as follows: winter wheat (*Triticum aestivum* subsp. *aestivum* L.), maize (*Zea mays* L.), maize, soybean (*Glycine max* L. Merr.), winter wheat, maize, and cowpea (*Vigna unguiculata* L. Walp). In 2018, on 15 m^2^ (5 m × 3 m) within each plot, an additional experiment was established to evaluate the effect of water stress on soil microbes and C and N pools under both CT and NT. Field operations and the entire set-up of the experiment are detailed in Guzzetti et al. [32]. Briefly, to test the effect of water stress, experimental plots were divided into two subplots at the early bloom stage of cowpea. The first one was sprinkler-irrigated three times (20 mm per time) to prevent water stress (NS: no stress), while the second subplot was temporarily covered by a self-constructed steel structure over the plants with a plastic sheet to avoid any natural or artificial water input, inducing water stress and simulating a very dry season (S: stress). These structures were of 1.8 m from the ground at their highest, and the covering sheet was regularly opened from its sides to allow airflow and to avoid overheating.

### 2.2. Analyses of Soil Microbial Diversity

The whole soil DNA was extracted using the DNeasy PowerSoil Kit (REF 12888–100, QIAGEN GmbH, Hilden, Germany) according to the manufacturer’s protocols. Bacterial diversity was analyzed using the V3-V4 region of the 16S ribosomal RNA (rRNA) gene, and fungal diversity of the samples was analyzed by sequencing the Internal Transcribed Spacer 1 (ITS1) genomic region of ribosomal DNA (rDNA), as previously described in detail [9,33,34]. Thermal cycling conditions and information-related primers and reagents of PCR amplification are detailed in Supplementary Table S1. Following the PCR, amplicons from the second steps were multiplexed as a single pool separately for bacteria and fungi. Agencourt AMPure XP kit (REF A63880, Beckman Coulter, Milan, Italy) were then used for the purification of the pool following the manufacturer’s protocols. High-throughput sequencing and statistical analyses were carried out as previously detailed [35,36]. Paired reads were assembled with the “pandaseq” script [37] and demultiplexed using Fastx toolkit. Sequences, that were chimeric with large homopolymers (≥10), that did not align with targeted regions, were removed using Mothur v.1.32.1 [38,39]. Mothur and R v4.0 [40] were used to analyze the final sequences by following two main approaches: the operational taxonomic unit (OTU) and the taxonomy-based. For the former, sequences were first aligned against the SILVA reference-aligned database for bacteria [41] using the NAST algorithm and a kmer approach [42,43] and then clustered at a 3% distance using the average linkage algorithm. Selected OTUs, having a sum of their abundances across all samples of less than 0.1% of the total were grouped into a single “rare OTUs” group. For the latter, Greengenes database was used to classify them into taxa [44]. Sequence data were submitted to the National Centre for Biotechnology Information Sequence Read Archive (BioProject ID PRJNA687154).

### 2.3. Enzymatic Activities and Soil Alteration Index

Soil β-Glucosidase (β-GLU, EC 3.2.1.21), phosphatase (PHO, E.C. 3.1.3.2), and urease (URE, E.C. 3.5.1.5) enzyme activities were determined on soil samples that were freshly sieved (<2 mm), then kept immediately at −20 °C until analyses. Assays to determine activities of the above-mentioned enzymes in soil samples were performed as previously described, in detail, by Eivazi and Tabatabai [45], Sannino and Gianfreda [46], and Kandeler and Gerber [47], respectively, and the methods used are detailed in Supplementary Table S2. Measured activities of these enzymes were then used to calculate the scores of Alteration Index 3 (AI3) of Puglisi et al. [16].

### 2.4. Aggregate-Sized C and N Fractions

Undisturbed soil samples for determining aggregate-sized associated C and N fractions were collected in November 2018, right after cowpea harvest, with a 5 cm-diameter auger to a depth of 20 cm. Each soil core was divided into 0–5-cm and 5–20-cm soil depths after extraction. Three random subsamples from each plot and soil depth were pooled together. Soil aggregate classes were determined according to Elliott [48]. Briefly, moist soil sample was sieved at 8 mm and air-dried. An 80-g subsample was submerged into deionized water for 5 min and wet sieved, obtaining four soil aggregate fractions: large macroaggregates (LM; >2000 µm), small macroaggregates (sM; 250 µm–2000 µm), microaggregates (m; 53 µm–250 µm), and silt and clay fraction (s + c; <53 µm). Fractions within macroaggregates were isolated according to Six et al. [49], thus obtaining coarse particulate organic matter (cPOM; >250 µm), microaggregates within macroaggregates (mM; 53 µm–250 µm), and silt and clay (s + cM; <53 µm). All fractions were corrected for sand content according to Kemper and Rosenhau [50]. One gram of soil per each fraction was then weighed and analyzed for C and N concentrations by the Dumas combustion method with an elemental analyzer (VarioMax C:NS, Elementar, Langenselbold, Germany).

### 2.5. Statistical Analyses

Data on soil C and N concentrations within each aggregate-sized fraction were subjected to analysis of variance (ANOVA) with a split-plot design following procedures outlined by Gomez and Gomez [51] and using the “agricolae” package of RStudio 3.3.3. The main plot factor was the tillage system (T), while the subplot factor was water availability (W). When the Shapiro-Wilk test did not confirm the assumptions of ANOVA, data were log-transformed before analysis. Tukey’s honestly significant difference (HSD) was used to test for significant differences in variables among treatments with a *p*-value of 0.05 as the threshold for statistical significance [51]. Indices of α-diversity that were used in this study were calculated with modules of Mothur, as detailed in Vasileiadis et al. [36]. Statistical analyses on the OTU and taxonomy matrices were performed in Mothur and R. This included hierarchical clustering with the average linkage algorithm at different taxonomic levels, principal component analysis (PCA) to assess the unconstrained sample groupings, and canonical correspondence analyses (CCA) to assess the significance of different treatments on the analyzed diversity. Metastats [52] was applied to identify features that were significantly different between treatments. Enzymatic activities and soil alteration index results were statistically analyzed by one-way analysis of variance (ANOVA) at a 95% confidence level. The means of treatments were statistically compared by the least significant differences (LSD) test using CoStat Statistical Software (Version 6400, CoHort Software, Monterey, CA, USA).

## 3. Results

### 3.1. Analyses of Soil Microbial Diversity

**Figure 1:**
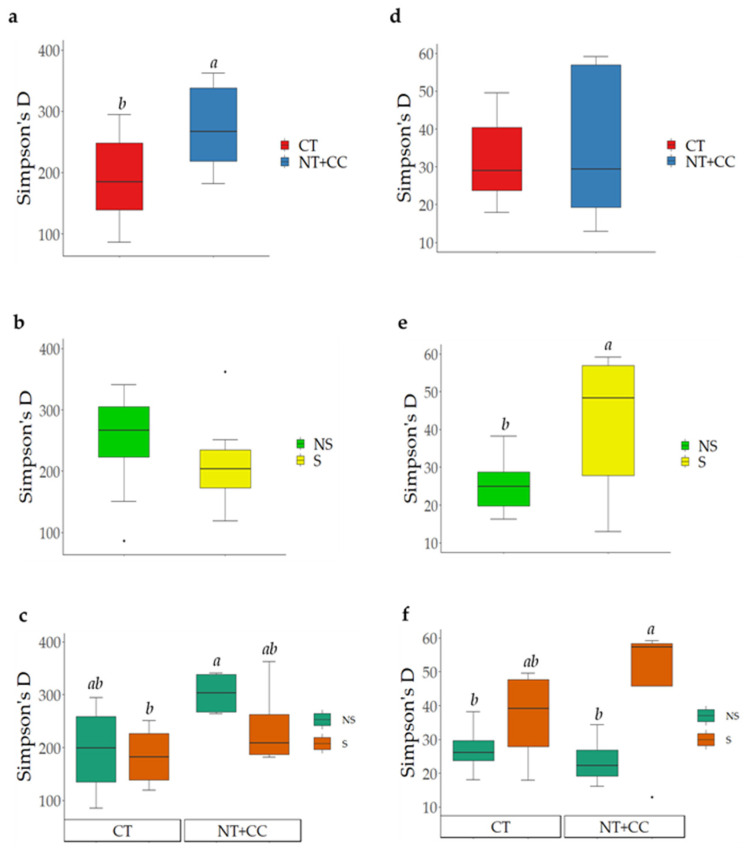
Biodiversity of bacteria (**a**–**c**) and fungi (**d**–**f**), as affected by the tillage, water stress conditions, and their interaction according to Simpson’s D (presence of letters indicate a statistical significance of *p* ≤ 0.05). (NT + CC: No-till and Cover Crops, CT: Conventional Tillage, S: Water Stress, and NS: No Water Stress).

The filtering, demultiplexing, and rarefaction of high-throughput sequencing (HTS) reads resulted in a common number of 50,000 reads per sample for both the 16S and ITS samples; the related average coverage was 97.5% for bacterial amplicons and 99.6% for fungal amplicons, thus indicating that the whole diversity of the microbial communities was covered. The bacterial diversity was significantly higher in NT + CC than in CT (Figure 1a), and stress did not cause significant changes on the bacterial diversity (Figure 1b). The NT + CC × NS interaction resulted in a significantly higher diversity than CT × S (Figure 1c). Tillage was insignificant for the changes in fungal diversity (Figure 1d). However, water stress significantly increased the fungal diversity (Figure 1e). The interaction of NT + CC × S resulted in the highest significant fungal diversity (Figure 1f).

**Figure 2:**
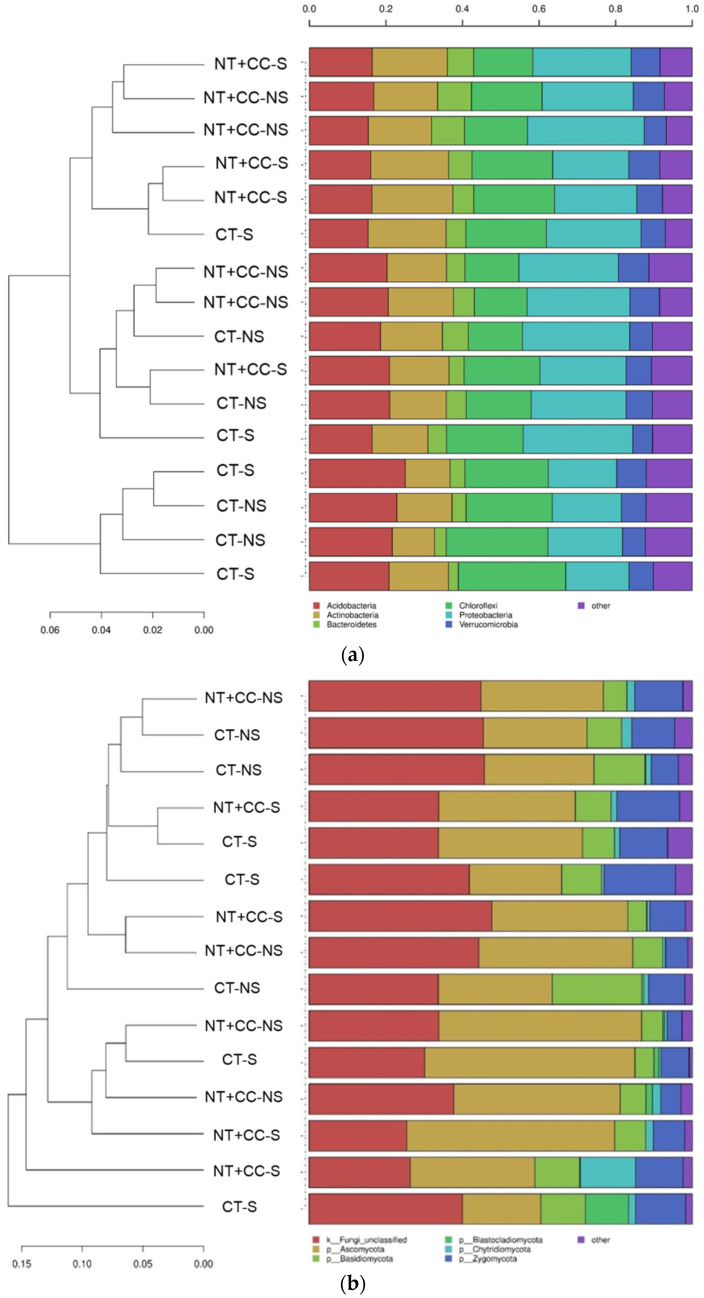
Taxonomic comparison of all samples ((**a**) bacteria and (**b**) fungi). Hierarchical clustering of microbial communities at the phylum level across all samples used in this study. Clusters were identified with the average linkage algorithm for taxa that contributed at least 5% to a single sample. Taxa that contributed less than this threshold were added to the sequence group denoted “other”.

A taxonomic comparison of the microbial communities at the phylum level across all samples is presented in Figure 2. The results indicated a heterogeneity across the experimental conditions of this study, especially for the soil bacterial communities (Figure 2a). The bacterial samples were predominated by *Proteobacteria* and, then, by *Acidobacteria*, *Chloroflexi*, and *Actinobacteria* phyla, regardless of treatment. The only exception was a group of CT-NS samples, which were predominated by *Chloroflexi*, followed by *Acidobacteria* and *Proteobacteria*. In the fungal communities (Figure 2b), the predominance of the phylum *Ascomycota* was evident in NT + CC conditions, with only a few exceptions. In comparison, unclassified fungi mostly dominated the communities in the samples from the CT plots. In both cases, *Basidiomycota* and then *Ascomycota* (Figure 2b) followed the dominant phylum.

**Figure 3:**
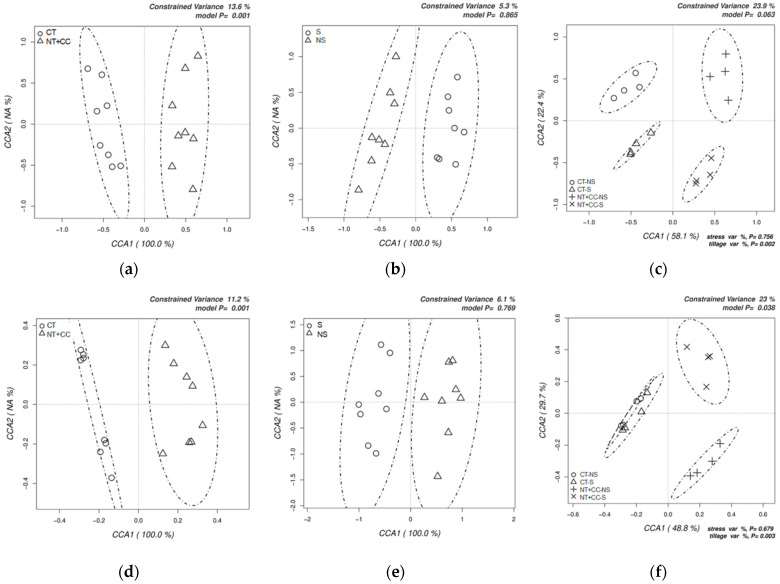
Canonical correspondence analyses (CCAs) on the impact of the tillage, stress, and their interaction on the structure of the bacterial upper half (**a**–**c**) and fungal lower half (**d**–**f**) communities. Determined by the relative abundances of all the operational taxonomic units (OTUs) obtained by Illumina sequencing of the bacterial 16S and fungal Internal Transcribed Spacer (ITS) amplicons.

Bacterial and fungal OTU abundance tables were subjected to a multivariate canonical correspondence analysis (CCA) to assess the possible significance of tillage, water, and their combined impact on the structures of the bacterial (Figure 3a–c) and fungal communities (Figure 3d–f). Tillage was most important factor for the clustering of the bacterial communities (Figure 3a). Water stress was insignificant (Figure 3b), whereas tillage and stress in combination formed four distinctive clusters (Figure 3c), indicating the impact of tillage on the soil bacteria, especially under water stress. Similarly, the tillage was the most significant factor (Figure 3d), and water stress alone did not have a significant impact (Figure 3e) on the fungal OTUs clustering. Two separate clusters of stress highlighted the combined impact conditions under NT + CC. Clusters of CT remained unaffected by the stress conditions (Figure 3f).

**Figure 4:**
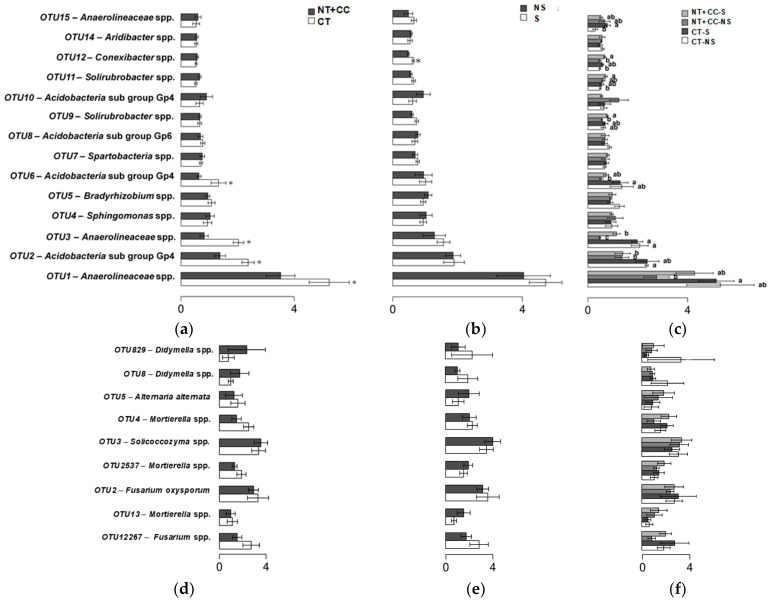
Bacterial (**a**–**c**) and fungal (**d**–**f**) OTUs as analyzed by a Metastats aimed at identifying the ones with significant differences (letters or *, *p* ≤ 0.05) between treatments of tillage (**a**,**d**), stress (**b**,**e**), and their interaction (**c**,**f**) at 0–4% intervals.

The Metastats analysis results showed that abundances of the following bacterial OTUs were significantly affected by tillage (Figure 4a): *Anaerolineaceae* spp. (OTU1, 3), *Acidobacteria* subgroup Gp4 (OTU2), and *Acidobacteria* subgroup Gp4 (OTU6). *Conexibacter* spp. (OTU12) abundance was significantly increased under stress conditions (Figure 4b). The following were significantly affected by various combinations of tillage and stress (Figure 4c): *Anaerolineaceae* spp. (OTU1, 3, and 15); *Acidobacteria* subgroup Gp4 (OTU2, 6, and 10); *Bradyrhizobium* spp. (OTU5); *Spartobacteria* spp. (OTU7); *Acidobacteria* subgroup Gp6 (OTU8); *Solirubrobacter* spp. (OTU9 and 11); and *Conexibacter* spp. (OTU12). None of the fungal OTUs were found to be significantly affected by any of the treatments (Figure 4d–f). Comparisons of the microbial communities across all samples at the class level are also presented with the Supplementary Materials (Supplementary Figure S1).

### 3.2. Enzymatic Activities and Soil Alteration Index

The activities of β-glucosidase (βGLU), phosphatase (PHO), and urease (URE) enzymes measured in the soils under cowpea cultivation, and the impact of tillage and water stress conditions together with their combinations, on the measured activities are reported in Table 1. The impact of tillage was assessed according to the soil alteration index score AI3 using enzymatic activities to calculate the index scores of the samples. CT soils scored significantly higher than the NT + CC soils. The results on the stressed plots were not significantly different when compared with no stressed ones, and the combination of T × W (Tillage + Water) treatments resulted in greater alteration scores for NS soils under CT than NS soils under NT + CC. When compared separately, in NT + CC soils, the activities of βGLU and PHO were significantly higher than CT soils. The activity of URE, although slightly lesser in tillage, remained unaffected. In the case of combined treatments, the highest activities of βGLU and PHO were measured in NT + CC and no stress conditions, and the impact of water stress was not significant.

### 3.3. Aggregate-Associated C and N

The C concentration in the LM fraction significantly increased under NT + CC in the 0–5-cm soil layer (Table 2), whereas the tillage management did not cause C enrichment in the other aggregate-sized fractions. The water stress conditions (W) and the interaction between tillage (T) and water stress did not affect the C concentration in the aggregates in the surface soil. Within macroaggregates, the C associated to coarse particulate organic matter (cPOM) and to microaggregates within macroaggregates (mM) in the 0–5-cm soil layer was affected by tillage, water stress conditions, and their interaction. In detail, a higher C concentration was detected: (i) under NT + CC than under CT and (ii) under no water stress (NS) than under stress (S).

The interaction of T × W was significant: NT-NS and CT-NS had the highest and the lowest C concentrations, while CT-S and NT-S were intermediates between the two. The aggregate-associated C in the 5–20-cm layer was not significantly affected by either tillage or the water stress conditions. Within macroaggregate-occluded soil fractions, at 5–20-cm soil depth, the only significant difference was observed in the s + cM, which showed a C depletion under NT + CC, compared with CT.

Soil N in the LM fraction was increased under NT + CC compared with CT in the 0–5-cm soil layer (Table 3). Considering the macroaggregate-occluded fractions, NT + CC showed a higher N concentration than CT in cPOM and s + cM, while NS led to a N increase only in the cPOM. For this fraction, the interaction T × W was significant: the highest N concentration was observed under NT-NS, followed by NT-S, CT-S, and CT-NS. At a 5–20-cm depth, the N concentration in the m fraction was increased under NT and NS; however, the interaction T × W was not significant. As for C, within macroaggregates, the only significantly difference was observed for s + cM, in which the N decrease under NT + CC occurred when compared with CT.

### 3.4. Correlations between the Microbiological and Physicochemical Properties

**Figure 5:**
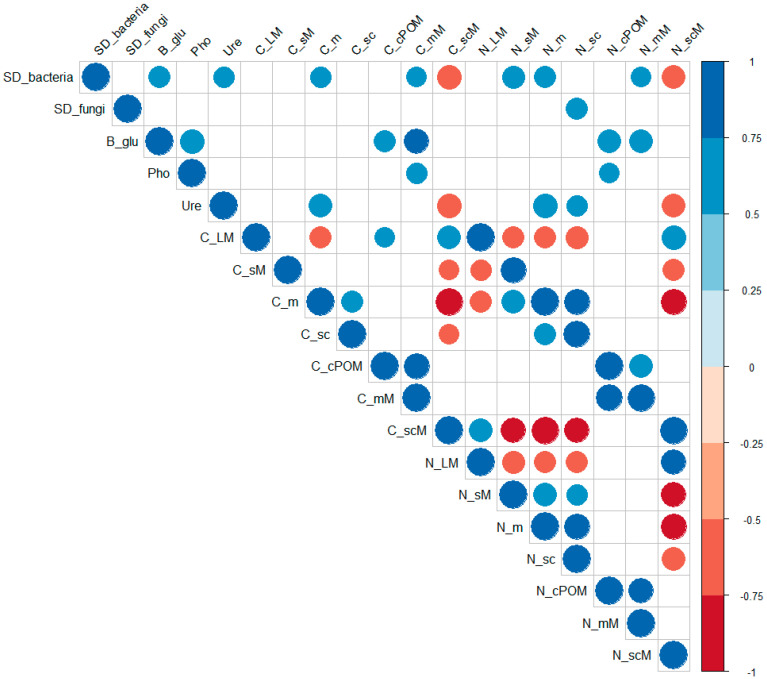
Pearson’s correlations among microbial and fungal diversity, enzyme activities, and C and N inclusion within soil aggregate-size classes. (SD_bacteria: Simpson’s diversity index of bacteria, SD_fungi: Simpson’s diversity index of fungi, β_glu: β-glucosidase activity, Pho: phosphatase activity, Ure: urease activity, C_LM: C in large macroaggregates (>2 mm), C_sM: C in small macroaggregates (2 mm–250 μm), C_m: C in microaggregates (250 μm—253 μm), C_sc: C in silt and clay (<53 μm), C_cPOM: C in coarse particulate organic matter (>250 μm), C_mM: C in microaggregates within macroaggregates (250–253 μm), C_scM: C in silt and clay within macroaggregates (<53 μm), N_LM: N in large macroaggregates (>2 mm), N_sM: N in small macroaggregates (2 mm–250 μm), N_m: N in microaggregates (250 μm–253 μm), N_sc: N in silt and clay (<53 μm), N_cPOM: N in coarse particulate organic matter (>250 μm), N_mM: N in microaggregates within macroaggregates (250–253 μm), and N_scM: N in silt and clay within macroaggregates (<53 μm)).

The Pearson’s correlation coefficient highlights the presence of a positive relation between the bacterial diversity (Bacterial Species Diversity (SD_bacteria)) (Figure 5) and C and N inclusion within m, mM and sM (the latest only for N), whereas the bacterial diversity and C and N concentrations within s + cM were negatively correlated. Considering the enzymatic activities, β GLU and URE were positively correlated with the bacterial diversity; a positive correlation was observed among β GLU, PHO, and C and N associated to cPOM and mM, while, for URE, this interaction was positive with C and N within m and negative with C and N associated to s + cM. The C and N concentrations associated to each aggregate-sized fraction showed significant positive correlations between them. In addition, C and N pools associated to LM were positively correlated with C and N in all within-macros fractions (i.e., cPOM, mM, and s + cM), even though these correlations were not always significant. Conversely, the C and N pools associated to sM, m, and s + c were (or tended to be, in some cases) negatively correlated with the C and N associated to LM.

## 4. Discussion

### 4.1. Analyses of Soil Microbial Diversity

Our results by both the α-diversity analyses and CCA showed that a combined impact of tillage and water stress significantly affected the soil bacteria and fungi. The increased α-diversity of the soil prokaryotic community, particularly soil bacteria, under NT + CC is in agreement with previous studies [53,54,55]. The heterogeneity of the soil microbial communities across the experimental conditions could be attributed to a stable community composition after seven years of continuous adaptation of NT + CC and to the fact that tillage may not induce significant shifts in abundances (Essel et al. [56]) or may cause only slight shifts of some groups, particularly *Anaerolineae* bacteria, as observed by Chavez Romero et al. under similar experimental conditions [57]. However, tillage can cause the formation of distinctive groups of microorganisms when coupled with other factors, as was also the case in our study [14]. A significantly higher fungal biodiversity under water stress conditions may be attributed to the fact that soil fungi coexist by occupying different moisture niches, acting as a sensitive indicator of water stress, as previously evidenced by Kaisermann et al. [58]. The importance of our results regarding the *Bradyrhizobium* spp. should also be highlighted, since *Bradyrhizobium* spp. are among important plant growth-promoting rhizobacteria (PGPR) [25]. Its abundance is generally affected by fertilization and rotations [1]. Our findings provided further evidence for their adaptability to water stress conditions and NT + CC management with a leguminous cowpea, as they remain unaffected by water stress and tillage. *Anaerolineaceae* are known users of carbon, especially under anaerobic conditions, and their abundance was found to be positively affected in the fields where the crop residues were abundant and often submerged in soil [21]. This can be explained by the findings of Fiorini et al. [20] from the same experimental field, in which increased soil porosity and oxygen concentration under NT + CC soils than under CT ones were found. *Solirubrobacter* was also found among the dominant bacteria in the soil under long-term tillage with cereals and legumes rotation by Essel et al. [56] and in the soils with more stable aggregates by Sánchez Marañón et al. [59].

### 4.2. Enzymatic Activities and Soil Alteration Index

The influence of soil tillage on the AI3 index was originally thought to be controversial [16]. However, the alteration status of CT soils is evidenced by changes in the microbial communities, enzymes, and the soil C/N in our study, and as a consequence, AI3 was able to confirm the altered status of CT, which is in accordance with the previous studies [60,61]. This was mainly driven by the effect of tillage on β-glucosidase activity, which was much higher under NT + CC than under CT soils. As β-glucosidase is directly involved in the metabolism of carbohydrates, the increase in β-GLU values under NT + CC could be explained with the high amount of residues left on the soil surface [62,63]. In addition, the positive effect of NT + CC on the phosphatase enzymatic activity can be explained by the effect of no-till to increase the P concentration in the topmost soil layers, as documented by Boselli et al. (2020) in the same experimental field. The differences between S and NS, especially under CT and NT, could be related to the fact that, under water stress, the activity and structure of the soil microbial communities are subject to change, eventually affecting the enzyme activities in soil related to processes they take apart [64]. The impact of water stress on the measured enzymatic activities were in accordance with previous findings for β-glucosidase [65] and for phosphatase [66,67].

### 4.3. Aggregate-Associated C and N

The C and N enrichment in LM due to NT + CC compared with CT (+172% and +129% for C and N, respectively) observed in our study in the surface soil layer (0–5 cm) confirms the crucial importance of reducing the soil disturbance for delaying the macroaggregate turnovers, thus enhancing the soil organic carbon (SOC) and soil total nitrogen (STN) stabilization [68]. In our experiment, the C and N concentrations in the NT + CC soil were mainly associated to macroaggregates (>250 μm), which accounted for around 80% of the total soil C and N. These results are consistent with previous findings by Lichter et al. [69] and Huang et al. [70], who found that C and N concentrations in macroaggregates under NT + CC range between 64% and 70% of the total SOC and between 56% and 60% of the STN. The stabilization of soil aggregates and the C enrichment under NT + CC in the shallow soil layer (0–5 cm) may be also positively affected by the increased root length and biomass of the main crops, which were previously detected in a three-year experiment in the same field [20]. Root exudates and root-sloughed cells are known to play a key role in the formation of soil aggregates, mainly in soils with high clay contents [71,72]. The effect of NT + CC in reducing aggregate turnovers is further corroborated by our results concerning the macroaggregate-occluded soil fractions. We indeed observed higher C and N concentrations in the cPOM fraction under NT + CC than under CT, confirming that cPOM plays as a core for macroaggregate formation [73,74]. At the same time, the increased C concentration in mM under NT + CC soils compared with CT ones shows the effective role of NT + CC for enhancing soil macroaggregates stabilization in the long term, since C-enriched mM were reported to be a sensitive indicator of decreased macroaggregate turnovers [49,74]. In addition, previous studies have highlighted the substantial role in SOC stabilization played by microaggregate-associated C [75,76]. Denef et al. [75] found that mM-associated C accounted for 20–47% of the total SOC content under NT + CC but explained 45–87% of the difference in SOC between the NT + CC and CT systems. Fiorini et al. [23] reported that this percentage could be even higher.

The rate of macroaggregates formation and the inclusion of C and N within them were also affected by the water availability under no-till in our study, with irrigated soils showing C and N enrichment compared with non-irrigated ones. Similar results were found by Smith et al. [77] in a 10-year experiment comparing NT + CC and CT under irrigated and dryland conditions on a silty-loam soil. Previous studies reported that water availability supports soil microbial processes involved in aggregate formation due to the release of microbial exudates derived from POM decomposition [73]. Therefore, the combination of NT + CC and NS led to the highest C increase within the macroaggregate-occluded fractions. On the contrary, under CT, water stress enhanced the C and N enrichment in the occluded fractions. According to Smith et al. [77], residue incorporation by tillage in dry conditions could sustain a larger population of microorganisms, which may increase the formation of organic exudates that stabilize aggregates but accelerates organic matter mineralization, with a consequent C loss. Below a 5-cm depth, the effects of tillage and water availability on soil aggregation and C and N inclusion within aggregates were less evident, as previously observed by Sainju et al. [78], Zhang et al. [79], and Wang et al. [80]. However, the tendency of C and N to be higher in the m fraction under NT + CC than under CT, as observed in our study, corroborates previous results by Lopez Bellido et al. [81], who found that C and N concentrations within microaggregates were significantly increased under NT + CC down to a 30-cm depth. This further confirms the role of NT + CC to prevent soil C and N losses also in the deeper soil layers. Moreover, the higher stabilization of C and N pools in soils under NT + CC compared with CT is further supported by our results on C and N associated to mM in the deeper soil layer. Instead, considering the s + cM fraction, while some authors [70,75] found a greater C concentration in this occluded fraction in NT + CC than in CT at the 5–20-cm soil depth, we observed that, in NT + CC, a C depletion occurred, compared with CT. Such a decrease could be related to the lesser sensitivity of s + cM-associated C to tillage disturbance, compared with the cPOM and mM fractions [70].

### 4.4. Correlations between the Microbiological and Physicochemical Properties

The positive correlations observed between bacterial diversity, enzymatic activity, and C and N enrichment in microaggregate soil fractions (m and mM) suggest that the abundance and the complementarity of bacteria species enhance the stabilization processes of C and N pools within microaggregates. Conversely, negative correlations between the microbial activity and (occluded) s + c-associated C and N were found. This agrees substantially with the classic aggregation models (e.g., Tisdall and Oades [82]) and corroborates the major role of bacteria for microaggregate formation [83]. As observed in our study, the activity of the enzymes involved in C and N cycling, such as β-glucosidase and urease, has a close relation with the microbial community structure and diversity. This was in agreement with previous findings reporting that the activity of β-glucosidase and urease are stimulated by the increase of SOC and microbial biomass that may represent a substrate to the microbes for enhancing the enzymatic activities. The presence of the organic substrate indeed activates soil microorganisms to produce enzymes involved in the nutrient cycles as a consequence of the increased supply of easily degradable soil organic matter [84]. In addition, our results highlight that the enzymatic activity involved in the metabolism of carbohydrates (i.e., β-glucosidase) could be considered as a main driver of the microaggregates formation and stabilization within macroaggregates. β-GLU was indeed found to be positively correlated with the cPOM- and mM-associated C and N pools in our study. Such a relationship suggests that the higher the amount of cPOM-associated C and N, the higher the β-glucosidase activity, which turns into increased C and N occluded into microaggregates. Thus, the results ultimately indicate the importance of the cPOM fraction as an energy source for microorganisms to stimulate the formation of new macroaggregates and the stabilization of C and N pools within them.

## 5. Conclusions

This study investigated the combined impact of long-term no-till (NT) and cover crops (CC) on soil bacterial and fungal communities, soil enzymatic activity, and the C and N accumulation in soil aggregates under different water stress conditions (NS (no stress) and S (stress)) that were introduced in the last year during the cultivation of *V. unguiculata*. NT + CC and S were the main driving factors for the changes in the soil biodiversity, especially for the bacteria. The microbial communities responded to tillage and stress by forming separate clusters, indicating the impact of even a short but intense water stress on these communities and further highlighting the specific OTUs of interest. The findings of this study indicate a significant C and N increase under NT + CC due to C- and N-rich large macroaggregates (LM) and microaggregates within macroaggregates (mM), highlighting the major role of those fraction for C and N changes, as induced by soil tillage. Water stress was a limiting factor for C and N inclusion within the aggregates.

The findings suggest that NT + CC is a promising alternative to CT for intensive agroecosystems due to its contribution to soil C and N stabilization while enhancing soil microbial communities and soil enzymatic activities.

## Figures and Tables

**Table 1:** The impact of tillage, stress, and their combinations on the measured enzyme activities of β-glucosidase (β GLU), phosphatase (PHO), and urease (URE) in the soils (standard error of the means are indicated within parenthesis before significance letters (a, ab, b) which separately indicate a statistical significance of *p* ≤ 0.05 in the corresponding sections).

			Measured Soil Enzymes		
Source of Variation		AI3 ^5^	β GLU (μmol PNG g^−1^ h^−1^)	PHO (μmol PNP g^−1^ h^−1^)	URE (μg urea g^−1^ h^−1^)
Tillage (T)	CT ^1^	−85.35 (±5.05) b	8.86 (±0.8) b	18.51 (±0.48) b	5.85 (±1.07) a
	NT + CC ^2^	−38.43 (±5.86) a	20.85 (±2) a	24.21 (±1.6) a	7.05 (±1.1) a
Water (W)	NS ^3^	−60.72 (±4.47) a	16.84 (±4.17) a	23.13 (±1.85) a	6.47 (±1.63) a
	S ^4^	−63.05 (±5.83) a	12.86 (±1.41) a	19.6 (±0.55) a	6.42 (±1.12) a
T × W	CT-NS	−96.14 (±3.63) b	8.06 (±0.81) b	19.04 (±0.53) ab	6.18 (±1.13) a
	CT-S	−74.56 (±4.65) ab	9.65 (±0.85) b	17.98 (±0.39) b	5.51 (±1.1) a
	NT + CC-NS	−25.3 (±3.33) a	25.63 (±1.51) a	27.21 (±1.4) a	6.77 (±2.52) a
	NT + CC-S	−72.09 (±5.38) ab	16.07 (±1.14) b	23.72 (±1.08) ab	7.33 (±1.99) a

^1^ CT: conventional tillage. ^2^ NT: no tillage. ^3^ NS: no stress. ^4^ S: stress. ^5^ Puglisi et al. [16]. AI3: Alteration Index 3. CC: cover crops.

**Table 2:** Soil total C content (g C kg^-1^ soil) of aggregate-sized fractions acquired from wet sieving of whole soil and from macroaggregates in different soil layers (0–5 cm and 5–20 cm) as affected by tillage (T), water (W), and interaction of T × W in different soil layers (0–5 cm and 5–20 cm).

Depth	Source of Variation	Code	Whole Soil—C Amount (g C kg^−1^ Soil)							Macroaggregates—C Amount (g C kg^−1^ Soil)			
			LM ^1^		sM ^2^	m ^3^	s + c ^4^	cPOM ^5^		mM ^6^		s + cM ^7^	
0–5 cm	Tillage (T)	CT	2.71	b	6.49	1.73	1.43	0.66	b	6.34	b	2.75	
		NT + CC	7.37	a	5.67	1.85	1.66	2.20	a	8.29	a	3.65	
		*p* value	0.0093		0.4249	0.5076	0.2816	0.0039		0.0433		0.0688	
	Water (W)	NS	6.20		6.59	1.77	1.63	1.66	a	7.59	a	3.55	
		S	3.88		5.57	1.81	1.45	1.19	b	7.03	b	2.85	
		*p* value	0.0779		0.2344	0.7912	0.3374	0.0401		0.0258		0.0676	
	T × W	CT-NS	3.74		5.52	1.63	1.57	0.43	c	6.27	b	2.93	
		CT-S	1.68		7.47	1.83	1.28	0.89	bc	6.40	ab	2.57	
		NT + CC-NS	8.68		5.62	1.91	1.69	2.90	a	8.92	a	4.18	
		NT + CC-S	6.07		5.72	1.79	1.62	1.50	b	7.65	ab	3.13	
		*p* value	0.8142		0.2800	0.3219	0.5429	0.0028		0.0109		0.3187	
5–20 cm	Tillage (T)	CT	4.59		5.24	1.42	0.89	0.57		5.97		3.84	a
		NT + CC	4.17		5.64	1.62	0.94	0.56		6.61		2.70	b
		*p* value	0.6124		0.4097	0.0636	0.5325	0.7415		0.0720		0.0396	
	Water (W)	NS	4.89		5.53	1.31	0.81	0.60		6.31		3.53	
		S	3.87		5.35	1.73	1.01	0.53		6.27		3.01	
		*p* value	0.1196		0.5502	0.0698	0.1453	0.5609		0.8356		0.0688	
	T × W	CT-NS	4.94		4.92	1.24	0.83	0.55		5.91		4.15	
		CT-S	4.24		5.57	1.59	0.94	0.60		6.02		3.52	
		NT + CC-NS	4.84		5.50	1.37	0.79	0.65		6.71		2.92	
		NT + CC-S	3.50		4.92	1.86	1.08	0.46		6.51		2.49	
		*p* value	0.5888		0.1531	0.6745	0.4593	0.3593		0.4858		0.6980	

^1^ LM: macroaggregates with a large size (>2 mm). ^2^ sM: macroaggregates with a small size (2 mm–250 μm). ^3^ m: microaggregates (250 μm–253 μm). ^4^ s + c: silt and clay (<53 μm). ^5^ cPOM: coarse particulate organic matter within macroaggregates (>250 μm). ^6^ mM: microaggregates within macroaggregates (250 μm–253 μm). ^7^ s + cM: silt and clay within macroaggregates (<53 μm). S: water stress. NS: no water stress. When present, letters (a, ab, b, bc, c) indicate a statistical significance of *p* ≤ 0.05.

**Table 3:** Soil total N contents (g N kg^−1^ soil) of the aggregate-sized fractions acquired from the wet sieving of whole soil and from macroaggregates in different soil layers (0–5 cm and 5–20 cm) as affected by tillage (T), water (W), and the interaction of T × W in different soil layers (0–5 cm and 5–20 cm).

Depth	Source of Variation	Code	Whole Soil—N Amount (g N kg^−1^ Soil)						Macroaggregates—N Amount (g N kg^−1^ Soil)					
			LM ^1^		sM ^2^	m ^3^		s + c ^4^	cPOM ^5^		mM ^6^		s + cM ^7^	
0–5 cm	Tillage (T)	CT	0.38	b	0.76	0.22		0.14	0.05	b	0.95		0.34	b
		NT + CC	0.87	a	0.65	0.21		0.16	0.17	a	0.79		0.51	a
		*p* value	0.0317		0.2986	0.9460		0.1566	0.0040		0.1375		0.0356	
	Water (W)	NS	0.73		0.78	0.20		0.16	0.12	a	0.88		0.45	
		S	0.51		0.63	0.23		0.15	0.09	b	0.87		0.40	
		*p* value	0.1188		0.1685	0.3011		0.5377	0.0426		0.6622		0.3658	
	T × W	CT-NS	0.45		0.62	0.19		0.14	0.03	c	0.78		0.36	
		CT-S	0.30		0.90	0.24		0.13	0.07	bc	0.80		0.32	
		NT + CC-NS	1.02		0.63	0.21		0.17	0.21	a	0.98		0.54	
		NT + CC-S	0.72		0.67	0.21		0.16	0.12	b	0.93		0.47	
		*p* value	0.5748		0.2753	0.3251		0.8816	0.0020		0.2588		0.7685	
5–20 cm	Tillage (T)	CT	0.58		0.62	0.17	b	0.12	0.04		0.75	b	0.49	
		NT + CC	0.53		0.71	0.21	a	0.13	0.05		0.88	a	0.38	
		*p* value	0.6322		0.2829	0.0479		0.5391	0.1422		0.0213		0.0547	
	Water (W)	NS	0.62		0.66	0.22	a	0.11	0.05		0.81		0.46	
		S	0.49		0.69	0.16	b	0.14	0.04		0.82		0.41	
		*p* value	0.1118		0.8928	0.0351		0.0998	0.4358		0.7583		0.2126	
	T × W	CT-NS	0.64		0.58	0.15		0.11	0.04		0.75		0.52	
		CT-S	0.52		0.68	0.19		0.12	0.04		0.75		0.45	
		NT + CC-NS	0.61		0.74	0.17		0.10	0.06		0.88		0.39	
		NT + CC-S	0.46		0.67	0.24		0.16	0.05		0.89		0.36	
		*p* value	0.8395		0.1653	0.5989		0.1403	0.3549		0.7353		0.6329	

^1^ LM: macroaggregates with a large size (>2 mm). ^2^ sM: macroaggregates with a small size (2 mm–250 μm). ^3^ m: microaggregates (250 μm–253 μm). ^4^ s + c: silt and clay (<53 μm). ^5^ cPOM: coarse particulate organic matter within macroaggregates (>250 μm). ^6^ mM: microaggregates within macroaggregates (250–253 μm). ^7^ s + cM: silt and clay within macroaggregates (<53 μm). S: water stress. NS: no water stress. When present, letters (a, ab, b, bc, c) indicate a statistical significance of *p* ≤ 0.05.

## Data Availability

Data available in a publicly accessible repository that does not issue DOIs. Sequence data were submitted to the National Centre for Biotechnology Information Sequence Read Archive (BioProject ID PRJNA687154).

## Supplementary Materials

The following are available online at https://www.mdpi.com/2079-7737/10/1/23/s1, Supplementary Figure S1: Hierarchical clustering of microbial communities at the class level across all samples used in this study. Supplementary Table S1: PCR reaction mixtures and thermal profiles for different target genes used. Supplementary Table S2: Detailed methodology used for the enzymatic assays of the soil samples.
