# Supplementary material for: Combined Impact of No-Till and Cover Crops with or without Short-Term Water Stress as Revealed by Physicochemical and Microbiological Indicators
Source: Biology (Basel). 2021 Jan 1;10(1):23. doi: 10.3390/biology10010023 (PMC7824270; doi:10.3390/biology10010023)
Supplement: Supplementary file 1 [file biology-10-00023-s001.pdf]

biology

MDPI

Supporting Information for the Article

Combined impact of no-till and cover crops with *or* without short-term water stress as revealed by physico-chemical and microbiological indicators

Eren Taskin[1], Roberta Boselli[2], Andrea Fiorini[2], Chiara Misci[1], Federico Ardenti[2], Francesca Bandini[1], Lorenzo Guzzetti[3], Davide Panzeri[3], Nicola Tommasi[3], Andrea Galimberti[3], Massimo Labra[3], Vincenzo Tabaglio[2*], Edoardo Puglisi[1],

* Correspondence: vincenzo.tabaglio@unicatt.it ; +39-0523-599-222 (V.T.)

biology

MDPI

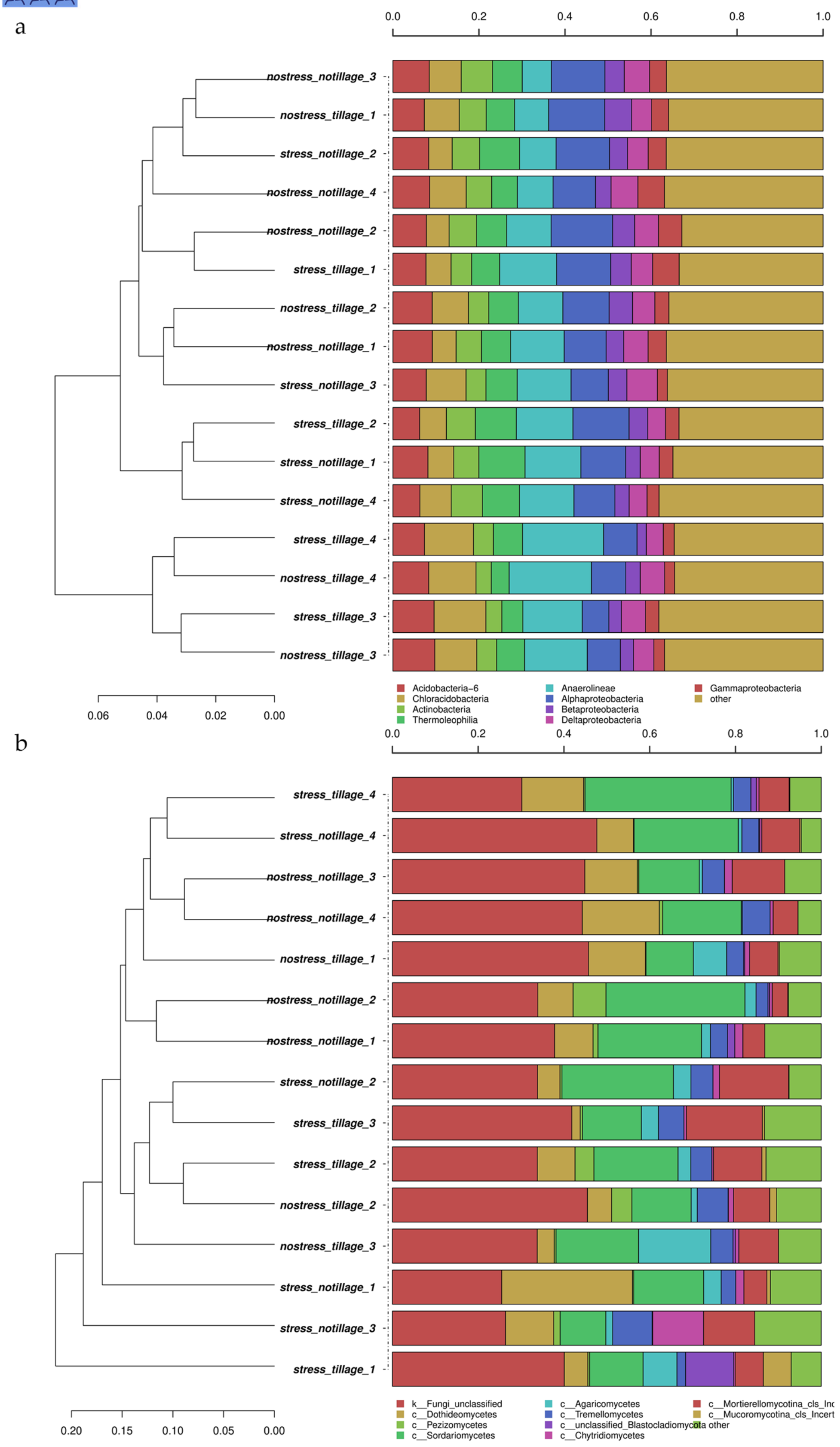

Supplementary Figure 1

biology MDPI

Supplementary Table 1. PCR reaction mixtures and thermal profiles for different target genes used

Target Gene	Reaction Mix	Volume (µL)	Step 1
Bacterial 16s 1st step	Phusion Flash High-Fidelity Master Mix	12.5	94 °C - 5 min<br>20x {94°C 30s, 50°C 30s, 72°C 30s}<br>72 °C 10 min.
	Nuclease free water	8	
	DNA template (1ng/µL)	2	
	Primer 343F (10µM) (5′-TACGGRAGGCAGCAG-3′)	1.25	
	Primer 802R (10µM) (5′-TACNVGGGTWTCTAATCC-3′)	1.25	
Bacterial 16s 2nd step	Phusion Flash High-Fidelity Master Mix	12.5	94 °C - 5min<br>10x {95°C 30s, 50°C 30s, 30°C 30s}<br>72 °C 10min.
	Nuclease free water	8	
	1st Step Amplicons	1.25	
	Primer 343F (10µM) (5′-TACGGRAGGCAGCAG-3′)	1.25	
	Primer 802R (10µM) (5′-TACNVGGGTWTCTAATCC-3′)	1.25	
Fungal ITS 1st step	Phusion Flash High-Fidelity Master Mix	12.5	94 °C - 4 min<br>25x {94°C 30s, 56°C 30s, 72°C 1min}<br>72 °C 7min
	Nuclease free water	8	
	DNA template (1ng/µL)	1.25	
	Primer ITS-1 (10µM) (5'-TCCGTAGGTGAACCTGCGG-3')	1.25	
	Primer ITS-2 (10µM) (5'-GCTGCGTTCTTCATCGATGC-3')	1.25	
Fungal ITS 2nd step	Phusion Flash High-Fidelity Master Mix	12.5	94 °C - 4 min<br>7x {94°C 30s, 56°C 30s, 72°C 1min}<br>72 °C 7min
	Nuclease free water	8	
	1st Step Amplicons	1.25	
	Primer ITS-1 (10µM) (5'-TCCGTAGGTGAACCTGCGG-3')	1.25	
	Primer ITS-2 (10µM) (5'-GCTGCGTTCTTCATCGATGC-3')	1.25	

Supplementary Table 2. Detailed methodology used for enzymatic assays of soil samples.

Enzyme	Incubated In	Incubation Conditions	Reaction Stopped Following Incubation by Adding	Activity Determined by
β-GLU	Buffered substrate solution (Modified Universal Buffer MUB pH 6.0 + 25mM 4-Nitrophenyl β-D-glucopyranoside)	Continuous shaking 250rpm, 37°C, 1h.	0.1M Tris-(hydroxylmethyl)-aminomethane pH 12 and 0.5M CaCl2 and vigorous shaking	Immediate separation of liquid phase and then spectrophotometrically at 405nm of liquid phase
PHO	Buffered substrate solution (MUB pH 6.5 + 25 mM p-nitrophenyl phosphatase)	Continuous shaking 250rpm, 37°C, 1h.	0.5M NaOH and 0.5M CaCl2 and vigorous shaking	
URE	Buffered substrate solution (Boric Buffer, pH 10 + 0.72M UREA)	Continuous shaking 250rpm, 37°C, 2h.	1N KCl/0.01N HCl solution and vigorous shaking	Liquid phase separated, then mixed with Sodium Salicylate solution (equal mix of 0.12% Na2Fe(CN)5NO, of 17% C7H5NaO3 and H20BD) and 1mL of 0.1% C3Cl2N3NaO3. Re-incubated at 24°C, 30 min and then spectrophotometrically determined at 690nm
